# 8-Oxoguanine DNA Glycosylase (OGG1) Deficiency Exacerbates Doxorubicin-Induced Cardiac Dysfunction

**DOI:** 10.1155/2022/9180267

**Published:** 2022-03-27

**Authors:** Chukwuemeka George Anene-Nzelu, Peter Yiqing Li, Tuan Danh Anh Luu, Shi Ling Ng, Zenia Tiang, Bangfen Pan, Wilson Lek Wen Tan, Matthew Ackers-Johnson, Ching Kit Chen, Yee Phong Lim, Rina Wang Miao Qin, Wee Woon Chua, Lim Xin Yi, Roger Sik-Yin Foo, Yusaku Nakabeppu

**Affiliations:** ^1^Cardiovascular Disease Translational Research Programme, Yong Loo Lin School of Medicine, National University of Singapore, Singapore; ^2^Genome Institute of Singapore, A∗STAR, Singapore; ^3^Montreal Heart Institute, Montreal, Quebec, Canada; ^4^Department of Medicine, University of Montreal, Quebec, Canada; ^5^Department of Paediatrics, Yong Loo Lin School of Medicine, National University of Singapore, Singapore; ^6^Khoo Teck Puat-National University Children's Medical Institute, National University of Singapore, Singapore; ^7^Division of Neurofunctional Genomics, Department of Immunobiology and Neuroscience, Medical Institute of Bioregulation, Kyushu University, Japan

## Abstract

Doxorubicin is an anthracycline widely used for the treatment of various cancers; however, the drug has a common deleterious side effect, namely a dose-dependent cardiotoxicity. Doxorubicin treatment increases the generation of reactive oxygen species, which leads to oxidative stress in the cardiac cells and ultimately DNA damage and cell death. The most common DNA lesion produced by oxidative stress is 7,8-dihydro-8-oxoguanine (8-oxoguanine), and the enzyme responsible for its repair is the 8-oxoguanine DNA glycosylase (OGG1), a base excision repair enzyme. Here, we show that the OGG1 deficiency has no major effect on cardiac function at baseline or with pressure overload; however, we found an exacerbation of cardiac dysfunction as well as a higher mortality in *Ogg1* knockout mice treated with doxorubicin. Our transcriptomic analysis also showed a more extensive dysregulation of genes in the hearts of *Ogg1* knockout mice with an enrichment of genes involved in inflammation. These results demonstrate that OGG1 attenuates doxorubicin-induced cardiotoxicity and thus plays a role in modulating drug-induced cardiomyopathy.

## 1. Introduction

Heart failure (HF) is a major cause of mortality and morbidity worldwide, posing a huge burden to public health systems in both developed and developing countries [1]. Despite the multifactorial nature of HF, oxidative stress is a common unifying feature, as the various pathologies that lead to HF are associated with increased generation of reactive oxygen species (ROS) [2]. ROS generation is particularly relevant in doxorubicin-induced cardiotoxicity [3], an anthracycline drug often used as a treatment of choice for various types of adult and paediatric cancers, including leukaemia, lymphomas, and solid tumours [3]. Despite the highly effective antineoplastic properties of doxorubicin, one major side effect that limits its clinical utility is the dose-dependent cardiotoxicity caused by the drug, which can lead to irreversible cardiomyopathy and subsequent HF [4, 5]. Indeed, it is estimated that up to 10% of patients on doxorubicin or its derivatives will develop delayed cardiac complications within ten years after completion of the therapy [3].

Doxorubicin disrupts protein complexes in the mitochondrial electron transport chain and also binds to the reductase domain of endothelial nitric oxide synthase, thereby increasing production of superoxide (O_2_^−^) in the cardiac cells [3]. Furthermore, doxorubicin has been shown to decrease the levels of the selenium-dependent glutathione peroxidase, an enzyme involved in detoxification of reactive oxygen metabolites with a subsequent accumulation of ROS [6]. The doxorubicin-induced increase in ROS in the cells leads to DNA damage, especially in mitochondrial DNA (mtDNA), lipid peroxidation of cellular and mitochondrial membrane, as well as impaired mitochondrial oxidative phosphorylation which ultimately leads to cell death by apoptosis and necrosis [7]. One of the most common DNA lesions produced by the increased ROS in the cells is the oxidation of guanine residues to 7,8-dihydro-8-oxoguanine (8-oxoguanine or 8-oxoG) [5]. Levels of 8-oxoG were found to be elevated in failing human hearts, suggesting that increased accumulation of DNA damage could be implicated in the pathophysiology of HF [2].

The primary enzyme responsible for the repair of 8-oxoG is the base excision repair (BER) enzyme, 8-oxoguanine DNA glycosylase (OGG1) and it excises 8-oxoG by cleaving the N-glycosidic bond of the lesion [2]. By preventing accumulation of DNA damage, especially in mtDNA, OGG1 is therefore critical in reducing mitochondrial fragmentation as well as improving mitochondrial function in muscle cells [8]. In addition, cardiac overexpression of human mitochondrial isoform of OGG1 (OGG1-2a) led to a reduction in mtDNA damage and cardiac fibrosis in a mouse pressure overload model of cardiac hypertrophy [9]. We therefore set out to study the role of OGG1 in pressure overload-induced hypertrophy and doxorubicin-induced cardiotoxicity, paying particular attention to the transcriptional changes induced by doxorubicin in both wild-type and *Ogg1* knockout (KO) hearts.

## 2. Material and Methods

### 2.1. Generation of *Ogg1* Knockout Mice

*Ogg1^+/-^* mice were previously established [10] and have been backcrossed to C57BL/6J for more than 20 generations. Age-matched male wild-type and *Ogg1^−/−^* (*Ogg1*-KO) mice obtained by mating *Ogg1*^+/-^ mice were used for these studies. Six-week-old mice were used for the transverse aortic constriction (TAC) experiments, while 8-month-old mice were used for the doxorubicin study. Genotyping was performed on mouse ear clippings to confirm wild-type or *Ogg1*-KO status, as also previously described [10] using the following primers for *Ogg1*: mgO5-1, GTTAAGCTTCAAACGTGCCTC; mgO3-1, GAAGGACTGTCCAGAAGCTA; and mpII5′-3, AAAGTCTCTCATTAGTATCCC. A single band corresponding to 780 base pair reflects wild-type mice, whereas *Ogg1*-KO mice display a single band corresponding to 675 base pair. Heterozygous mice reveal dual bands of 675 base pairs and 780 base pairs.

### 2.2. Animal Experiments

Animal experiments were performed under a license approved by the IACUC (National University of Singapore). Pressure overload TAC surgery was performed on adult 6-week-old mice. Doxorubicin administration was carried out on 8-month-old mice. Echocardiography to monitor cardiac function and dimensions was performed on the mice 6 weeks after TAC surgery and 2 weeks after intraperitoneal injection with 15 mg/kg doxorubicin using the Vevo 2100 (Visualsonics) instrument. Purified adult mouse left ventricular cardiomyocytes (CMs) were isolated using a previously established protocol [11].

### 2.3. Mouse Cardiac Pressure Overload Surgery (TAC: Transverse Aortic Constriction)

TAC surgery was performed as described [12]. Briefly, anaesthesia was induced in mice in an induction chamber followed by partial thoracotomy for better visualisation of the transverse aortic arch. Ligation of the transverse aortic arch was done around the aorta distal between the innominate and left carotid arteries with a 7.0 Prolene suture overlying a 27.5 G needle. The sham-operated mice went through a similar surgical procedure without ligation of the transverse aortic arch. Enrofloxacin (0.1 mg/kg/each day for 3 days) and buprenorphine (0.03 mg/kg/each day for 3 days) were given as antimicrobial and analgesic, respectively, and were administered postsurgery.

### 2.4. Doxorubicin Administration

Eight-month-old wild-type and *Ogg1*-KO mice were randomly assigned to either the control group or the doxorubicin treatment group. Doxorubicin HCl (Sigma-Aldrich, Cat#: D1515,) was dissolved in 0.9% saline and administered by intraperitoneal injection at a dose of 15 mg/kg of body weight [13]. The control mice received injections of saline of comparable volume.

### 2.5. Quantification of mtDNA Damage

Total DNA was isolated from adult mouse cardiomyocytes from the different treatment groups using the Purelink Genomic DNA extraction kit. 20 ng of extracted DNA was used for each reaction. mtDNA damage was detected by quantitative PCR as previously described [14]. Briefly, two fragments (117 bp and 10 kb) are amplified from the mtDNA with primers listed in Supplementary Table 1. As DNA lesions are likely distributed randomly, it is expected that the amplification of the 10 kb will be randomly inhibited. After 20 amplification cycles, which should still be during the logarithmic phase of the PCR, the PCR mixture was separated on an agarose gel stained with SYBR Safe DNA gel stain (Thermo Fisher Scientific, Cat# S33102) and imaged (Gel Logic 200 imaging systems). PCR products were quantified by densitometric analysis using the ImageJ (a free software from NIH). The linearity of the reaction was confirmed by including a control reaction containing 50% template DNA. The ratio between 10 kb and 117 bp was used to give an estimate of mtDNA damage [14].

### 2.6. Immunohistochemistry

Heart samples were fixed in 4% paraformaldehyde for 24 hours, then embedded in paraffin, and sectioned at 4 *μ*m intervals. Paraffin samples were further treated with xylene (to remove paraffin) and rehydrated. Haematoxylin and eosin (HE) was applied to observe myocyte architecture and Masson trichrome (MT) to identify cardiac fibrosis. Quantification of fibrosis was calculated as the blue-stained areas relative to total ventricular area, using the ImageJ (NIH).

### 2.7. Cell Culture Experiments

Murine cardiomyocytes (CMs) and cardiac fibroblasts (CF) were obtained from 6-week-old wild-type mice and cultured as previously described [11]. Expression of *Ogg1* mRNA was measured by reverse-transcription and quantitative real time PCR (RT-qPCR) in CMs exposed to oxidative stress (H_2_O_2_) and hypoxia (0.2% oxygen environment), as well as CFs in the heart exposed to profibrotic transforming growth factor beta 1 (TGF-*β*1) and proinflammatory interleukin-1*β* (IL-1*β*). TGF-*β*1 (10 ng/ml) and IL-1*β* (10 ng/ml) were purchased from PeproTech (NJ, USA). Experiments were conducted in serum-free conditions and treatments were applied for 24 hours.

### 2.8. RNA Extraction and RT-qPCR

Total RNA was prepared using TRIzol (Thermo Fisher Scientific, Cat#:15596018) according to the manufacturer's protocol. cDNA was synthesized using the qScript™ cDNA synthesis kit (Quanta Biosciences, Cat#:95049). QPCR analysis was performed using gene-specific primers, PerfeCTa SYBR® Green Fast Mix (Quanta Biosciences, Cat#95047), and Roche Lightcycler apparatus and software. Oligonucleotide primers were designed using PrimerBLAST (NCBI) and listed in Supplementary Table (ST) 1, *18* s was used as housekeeping.

### 2.9. RNA-Sequencing Analysis

Two sets of RNA-sequencing were performed. For the first set, total RNA was extracted from ventricular CMs from 2 biological replicates of 4-week-old mice, 2 *Ogg1*-KO and 2 wild type. For the second set, total RNAs were prepared from ventricular CMs from 2 biological replicates each of the following groups: wild-type mice injected with normal saline (WT saline), *Ogg1*-KO injected with normal saline (KO saline), wild-type mice treated with doxorubicin (WT DXB), and *Ogg1*-KO mice treated with doxorubicin (KO DXB). Paired-end libraries were made using TruSeq kits (Illumina). The libraries were sequenced on the HiSeq2000, generating 2 × 100-bp paired-end reads. Reads were mapped against the mouse genome using the TopHat version 2.0.11 with default parameters. Counting reads for each gene were computed using htseq-count [15]. Differential gene expression analysis was performed using edgeR [16]. A gene is considered differentially expressed between different groups if the fold change is greater than log_2_ 0.5 and the *P* value is less than 0.05. Gene expression heat map was generated using R (version 3.1.2). Gene ontology analysis was performed by the AMIGO gene ontology [17].

### 2.10. Statistical Analysis

All animal in vivo experiments were performed with at least *n* = 3 biological replicates. Animal experimental groups were controlled for mouse age and sex. Student's *t*-test was performed to test for significance between the two groups. *P* value of less than 0.05 was considered significant.

## 3. Results

### 3.1. Expression of *Ogg1* mRNA in the Mouse Heart

**Figure 1:**
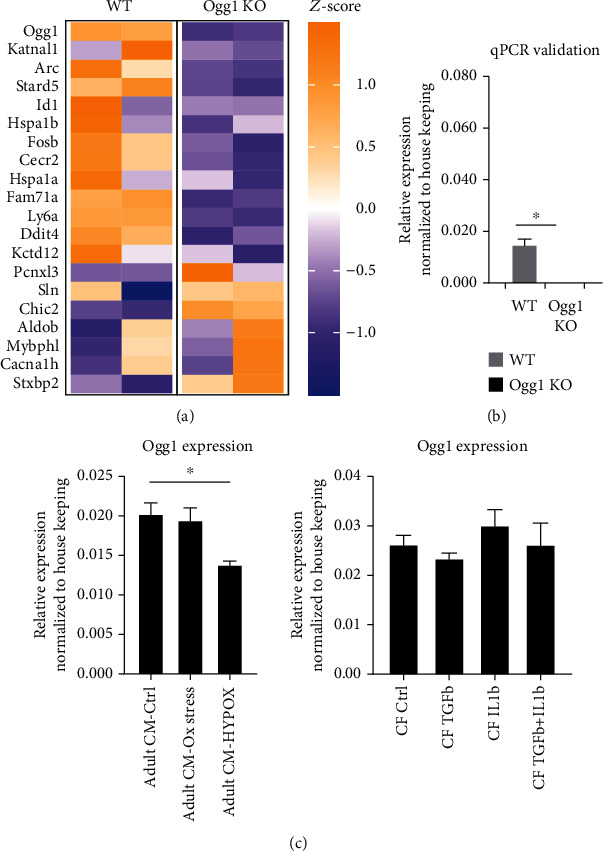
Expression of *Ogg1* in cardiomyocytes and cardiac fibroblasts at baseline. (a) Heat map showing the transcriptomic differences in the cardiomyocytes (CM) of wild-type (WT) mice compared to *Ogg1*-KO mice at baseline identified through RNA-sequencing analysis with a few genes highlighted. Z-scores are computed on a gene-by-gene basis and obtained by subtracting the mean and dividing by standard deviation. (b) qPCR validation of *Ogg1* mRNA expression in wild-type (WT) and *Ogg1*-KO CMs. All values are mean ± SE. *n* = 3^∗^*P* < 0.05. (c) Expression of *Ogg1* mRNA evaluated by qPCR in cardiomyocytes (CM) subjected to oxidative stress (Ox stress) and hypoxia (HYPOX), as well as cardiac fibroblasts (CF) in the heart treated with different stimuli. All values are mean ± SE. *n* = 3^∗^*P* < 0.05.

We first set out to evaluate *Ogg1* mRNA levels in mouse CMs by performing RNA sequencing and RT-qPCR on freshly isolated CMs from 4-week-old wild-type and *Ogg1*-KO hearts. Our results revealed a relatively low abundance of *Ogg1* mRNA in the wild-type CMs with fragments per kilobase of exon per million mapped reads (FPKM) value of ~2. In comparison, the FPKM level of cardiac-specific gene *Actc1* was 8000. *Ogg1* mRNA was however significantly depleted in the *Ogg1*-KO CMs,and also validated by RT-qPCR (Figures 1(a) and 1(b)). *Ogg1*-KO had very minor impact on the cardiac transcriptome at baseline as very few genes were significantly differentially expressed between wild-type and *Ogg1*-KO CMs (Figure 1(a), Supplementary Table 2). Next, we isolated adult CMs and CF from the wild-type heart, plated them in vitro, and exposed the cells to different stimuli to assess changes in *Ogg1* mRNA under different conditions. Our results showed that though *Ogg1* mRNA was slightly higher in the CF fraction, they were maintained relatively stable when exposed to different stimuli and only showed minimal downregulation in hypoxia in CM (Figure 1(c)).

### 3.2. Evaluation of Long-Term Effect of OGG1 Deficiency on Baseline Cardiac Function

**Figure 2:**
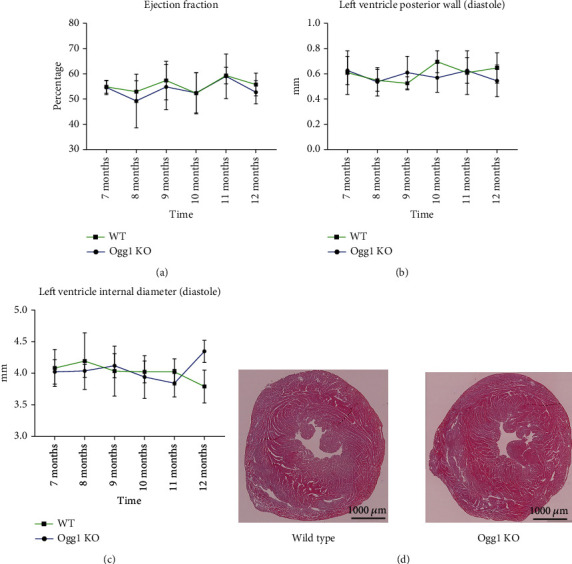
Heart morphology and function in aged WT and *Ogg1*-KO mice. (a) Ejection fraction, (b) left ventricular posterior wall, and (c) Left ventricle internal diameter measured monthly in both WT and *Ogg1*-KO mice from 7 months of age till 12 months. 3 mice per group. (d) Haematoxylin and Eosin staining of representative heart slices of WT and *Ogg1*-KO mice at 12 months of age.

OGG1 deficiency has been associated with the development of insulin resistance and obesity in mice with increasing age (12-15 months) [18]. We therefore assessed if OGG1 deficiency affects cardiac function over time. Using echocardiography, we monitored cardiac function by measuring the ejection fraction expressed in percentage (EF), left ventricular internal diameter (LVID), and left ventricular posterior wall (LVPW) in both *Ogg1*-KO and wild-type mice every month from the 7^th^ to 12^th^ months of age. No significant differences in the parameters were observed (Figures 2(a)–2(c)). Furthermore, there were no significant histopathological differences, comparing between *Ogg1*-KO and wild-type hearts (Figure 2(d)). OGG1deficiency therefore did not appear to result in any detectable difference in cardiac dysfunction with ageing. This lack of functional cardiac phenotype in *Ogg1*-KO mice is consistent with other studies that also reported no significant changes in cellular function over time in different cells of OGG1-deficient mice at baseline even up to 23 months [19, 20].

### 3.3. Effect of OGG1 Deficiency on Cardiac Function in a Mouse Model of Cardiac Hypertrophy Induced by Pressure Overload

**Figure 3:**
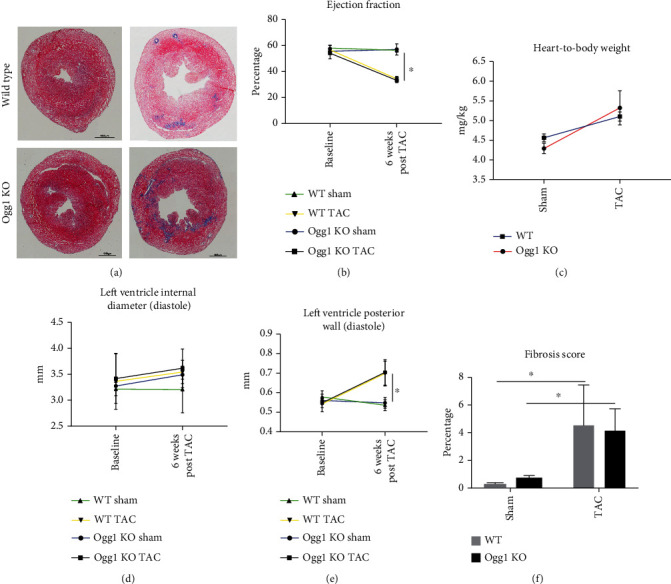
Cardiac function after transverse aortic constriction in WT and *Ogg1-*KO mice. (a) Representative heart sections stained with haematoxylin and eosin in WT and *Ogg1-*KO mice hearts to show left ventricle wall thickness and fibrosis after TAC surgery. (b) Ejection fraction, (c) heart weight-to-body weight, (d) left ventricle internal diameter, (e) left ventricle posterior wall, and (f) fibrosis score were assessed presurgery and 6 weeks post-TAC in WT and *Ogg1*-KO mice. All values are mean ± SD. *n* = 3 − 5^∗^*P* < 0.05.

To assess the role of OGG1 on cardiac function under cardiac stress, we subjected both wild-type and *Ogg1*-KO mice to TAC surgery to induce pressure overload. Both groups of mice showed a reduction in EF and an increase in the thickness of the ventricular walls after the TAC surgery compared to the sham-operated mice. There were no significant differences in EF, LVID, and LVPW between wild-type and *Ogg1*-KO hearts with TAC surgery (Figures 3(a)–3(e)). Similarly, no significant differences in fibrosis were seen (Figure 3(f)). While this is the first study to perform TAC surgery in OGG1-deficient mice, an earlier study had analysed the effect of human mitochondrial OGG1 (OGG1-2a) overexpression on cardiac function with TAC surgery [9]. In the earlier study, there were no differences in EF and wall dimensions between wild-type and the *OGG1* transgenic mice subjected to TAC surgery; however, there was a reduction in fibrosis in the *OGG1* transgenic mice after TAC surgery. We however did not observe any differences in fibrosis in our OGG1-deficient mice.

### 3.4. Effect of OGG1 Deficiency on Cardiac Function and Mortality after Doxorubicin (DXB) Treatment

**Figure 4:**
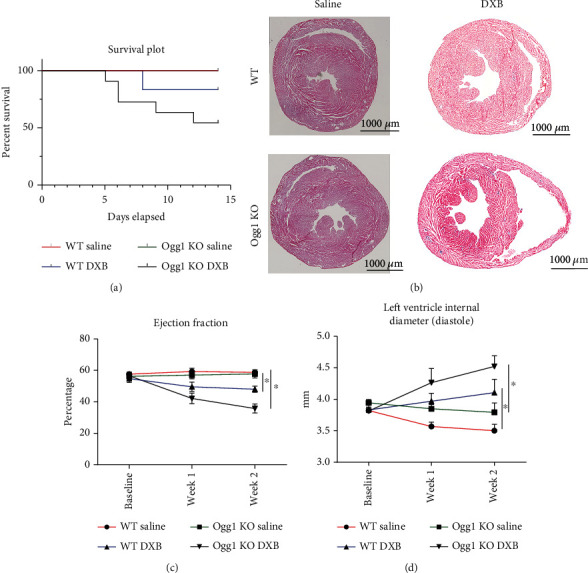
Cardiac function after doxorubicin treatment in WT and *Ogg1*-KO mice. (a) Survival plot 14 days after WT and KO mice were treated with 15 mg/kg doxorubicin. (b) Representative heart sections stained with haematoxylin and eosin 2 weeks after treatment with doxorubicin in both WT and *Ogg1*-KO mice. (c and d) Ejection fraction and left ventricle internal diameter measured at baseline, 1 and 2 weeks postdoxorubicin treatment. All values are mean ± SD. *n* = 3 − 7^∗^*P* < 0.05.

**Figure 5:**
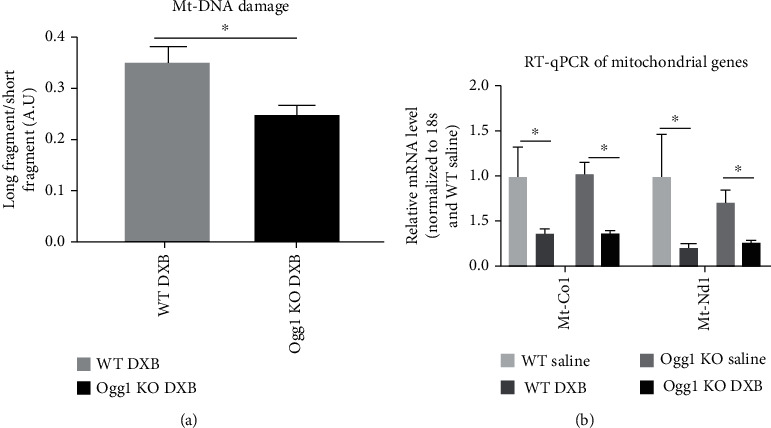
Mitochondrial DNA damage after doxorubicin treatment in WT and *Ogg1-*KO mice. (a) Mitochondrial DNA damage assessed after 2 weeks in doxorubicin-treated WT and *Ogg1-*KO mice. ^∗^*P* < 0.05. (b) qPCR of 2 mitochondrial genes quantified 2 weeks after treatment with doxorubicin. All values are mean ± SD. *n* = 3 − 7^∗^*P* < 0.05.

DXB induces oxidative stress in CMs with increased accumulation of 8-oxoG and mtDNA damage [5]; furthermore, DNA damage accumulates over time in different organs including the heart after DXB treatment [21]. We therefore examined the effect of DXB on the hearts of aged wild-type and *Ogg1*-KO mice (8-month-old mice). Mice received a single dose of 20 mg of DXB per kg of body weight and their cardiac function was analysed 2 weeks after the administration. First, we observed that about 50% (5 out of 10 mice) of *Ogg1*-KO mice died within 2 weeks after the DXB administration, while only 16% (1 out of 6 mice) of wild-type mice died within the 2 weeks (Figure 4(a)). *Ogg1*-KO mice that survived 2 weeks after the DXB administration exhibited a significantly greater EF reduction compared to wild-type mice, accompanied by significant LV dilatation (Figures 4(b) –4(d). DXB mediated LV dilatation is consistent with earlier reports [13], and this was more significant in *Ogg1*-KO mice. We next examined the level of mtDNA damage through PCR quantification of the ratio of long to short fragment. There was significantly greater mtDNA damage in the DXB-treated *Ogg1*-KO hearts compared to wild type (Figure 5(a)). Levels of mtDNA genes were however depleted equally in both wild-type and *Ogg1*-KO hearts upon treatment with DXB, an indication of mtDNA (Figure 5(b)) damage induced by DXB [22, 23].

### 3.5. Cardiac Transcriptomic Changes Induced by OGG1 Deficiency and DXB Treatment

**Figure 6:**
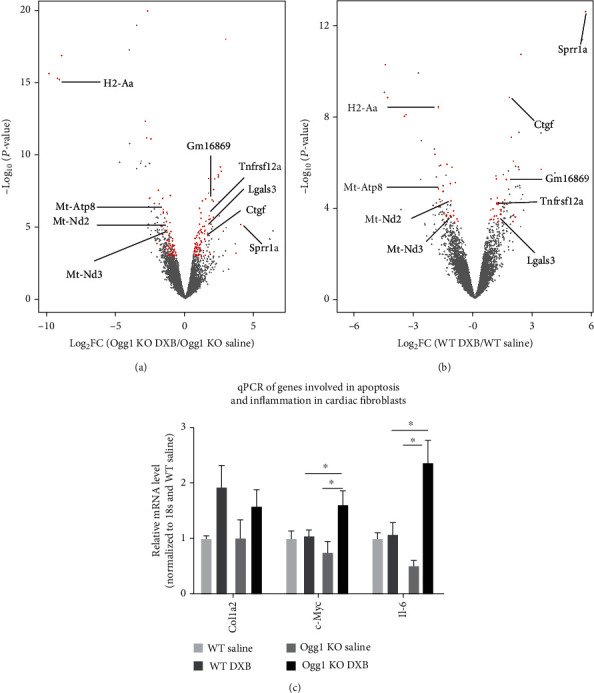
Transcriptomic profile after doxorubicin treatment in WT and *Ogg1*-KO mice. (a and b) Volcano plot showing genes that are changing after treatment with doxorubicin. The red dots are genes with fold change of log_2_ ≥ 0.5 and *FDR*value < 0.1. Some genes are highlighted. (c) qPCR of *Col1a2*, *c-Myc*, and *Il-6* in cardiac fibroblasts in the heart after treatment with doxorubicin.

Finally, we examined transcriptomic changes induced by DXB treatment by performing RNA sequencing in the four groups: wild-type and *Ogg1*-KO CMs treated with normal saline as the control, or with DXB. First, we analysed for differences between *Ogg1*-KO and wild-type hearts treated with normal saline. Interestingly, despite absent visible phenotypic changes in *Ogg1*-KO hearts over time, there were 270 differentially expressed genes (Supplementary Table 3). Gene ontology of the biological processes in upregulated genes showed terms such as “MHC Protein complex assembly”, Cellular Response to interferon beta and gamma”, and “positive regulation of cell killing” (Supplementary Table 4). These terms point to an activation of immune system, consistent with previous studies showing that the DNA damage response induces interferon signalling and activates immune response [24–26]. Gene ontology terms in downregulated genes included processes such as monocarboxylic acid metabolic processes (Supplementary Table 5). Next, we analysed DXB-induced changes compared within each group (*Ogg1*-KO: DXB vs. saline; and wild type: DXB vs. saline). There were more dysregulated genes in *Ogg1*-KO (DXB vs. saline; 465 genes) compared to wild type (doxorubicin vs. saline; 284 genes) (Figures 6(a) and 6(b), Supplementary Tables 6 and 7), with an overlap of 70 genes, of which 38 were upregulated and 32 were downregulated (Supplementary Table 8). One of the most significantly upregulated gene in both conditions was the *Sprr1a* gene which is a gp130 pathway- and stress-inducible gene [27]. Gp130 acts as mediator for the IL-6 family of cytokines and is involved in the regulation of inflammation and cardiac stress [27]. Other shared upregulated genes included *Ctgf*, *Lgals3*, and *Tnfrsf12a*. These genes have been shown to be predictive of cardiac dysfunction and cell death [28]. Among downregulated genes were a number of mitochondrial genes, downregulated in both *Ogg1*-KO and wild-type mice treated with DXB, including *Mt-Atp8*, *Mt-Nd2*, *Mt-Co3*, *Mt-Nd3*, and *Mt-Nd4*. This observation is consistent with previously published studies on mtDNA damage induced by DXB [5]. Indeed, GO terms for commonly dysregulated genes included terms “ATP synthesis coupled electron transport”, “Reactive oxygen species metabolic process”, and “Cellular respiration” confirming the effect of DXB on mitochondrial respiration (Supplementary Table 8). To shed light on the added effect of OGG1 deficiency on the DXB-treated CMs, we first focused on the uniquely dysregulated genes in the *Ogg1*-KO CMs treated with DXB (KO-DXB). Similar to the results found in the saline-treated *Ogg1*-KO CMs, there was enrichment for terms such as response to interferon gamma and response to cytokines, further showing that the DNA damage due to the OGG1 deficiency may lead to increase in inflammatory cytokines (Supplementary Table 9). Finally, we compared the transcriptomic changes in the DXB-treated *Ogg1*-KO CMs (KO-DXB) to DXB-treated wild-type CMs (WT-DXB), where we observed 61 upregulated and 68 downregulated genes in the KO-DXB compared to WT-DXB (Supplementary Table 10). GO for dysregulated genes showed enrichment for Notch signalling (Supplementary Table 11). Indeed, there was upregulation of Notch ligands *Jag1* and *Dll4* as well as notch target gene *Hey-1* [29]. These genes have also been found to be involved in the regulation of P53, a tumour suppressor often upregulated in response to DNA damage [30].

Given that OGG1 deficiency in the *Ogg1*-KO mice was global, we also assessed the level of other injury-associated genes in CFs. In particular, *Il6* and *Myc* were upregulated in the DXB-treated CFs harvested from *Ogg1*-KO mice (KO-DXB), compared to those from wild-type mice (WT-DXB) (Figure 6(c)). *Il6*, has been shown to be upregulated by DXB and is highly correlated with HF severity and cardiac remodelling [31]. Similarly, proapoptotic *c-Myc* was also shown before to be upregulated by DXB [32]. These results suggest that the impact of the OGG1 deficiency on cardiac homeostasis is mediated by both CFs and CMs.

## 4. Discussion

The role of OGG1-mediated base excision repair (BER) in the pathophysiology of oxidative stress-induced damage in different tissues has been the subject of many foregoing studies [19, 20]. In the cardiovascular system, the function of OGG1 has been analysed in ischemia reperfusion injury [14], TAC [9], and in atherosclerosis [33, 34], using gene knockout or overexpression approaches. Furthermore, OGG1 has been implicated in the development of DXB-induced cardiomyopathy in *Sirt3*-KO mouse model [5], in the prevention of mtDNA damage after abdominal aortic constriction [35], in the diabetic cardiomyopathy in mice [36], and in the end-stage cardiomyopathy in humans [2], implying that it may have an overall role in cardiac homeostasis. For all studies involving CMs, OGG1 levels correlate with levels of DNA damage but not always with cardiac function. Indeed a study found that despite increased DNA damage in *Ogg1*-KO mice, there was no evidence of mitochondrial dysfunction or cardiac dysfunction in *Ogg1*-KO mice [20]. In our study, cardiac EF in *Ogg1*-KO mice was not significantly perturbed up to 12 months of age. Similarly, there were no significant differences in cardiac function between the *Ogg1*-KO and wild-type mice after TAC surgery. These results suggest therefore that OGG1-mediated BER may not be critical for proper stress response of CMs. Indeed, expression of *Ogg1* mRNA in adult CMs is relatively low with FPKM values ranging from 1 to 2 in RNA sequencing data and its expression is not altered in TAC [12].

Nevertheless, systemic administration of DXB led to a more pronounced reduction in EF and increase in chamber dilatation in the DXB-treated *Ogg1*-KO mice, compared to wild type. There was also higher mortality in *Ogg1*-KO mice and larger number of dysregulated genes in the DXB-treated *Ogg1*-KO mouse hearts, suggesting that OGG1-mediated BER may ameliorate the DXB-induced cardiotoxicity. Our RNA sequencing data revealed an activation of the inflammatory pathway in *Ogg1*-KO mice. This is consistent with an earlier report that demonstrated that OGG1 deficiency led to increased inflammatory response in the lungs of mice exposed to hyperoxia [37]. They observed that the bronchioalveolar lavage fluid from the lungs of *Ogg1*-KO mice contained higher levels of inflammatory cytokines such as IL-6 and IFN-*γ* [37].

As DXB administration in our study was systemic, and the OGG1 deficiency was global in *Ogg1*-KO mice, we cannot rule out that the striking phenotypic and transcriptomic changes observed may have arisen in part due to signalling from other OGG1-deficient cells and tissues. Indeed, OGG1 deficiency has been shown to lead to increased NLRP3 inflammasome activation in macrophages, increased IL-1*β* production, and increased apoptosis in atherosclerotic plaques [34]. Nevertheless, our study provides the first report of DXB treatment in *Ogg1*-KO mice and also provides a transcriptomic analysis of both the impact of DXB on CMs as well as the potentiating effect of OGG1 deficiency on the cardiac injury produced by the drug. Further studies will be aimed at unravelling the mechanism through which OGG1 deficiency exacerbates cardiac dilatation in the DXB-treated hearts as well as the role of OGG1 in CFs.

## Data Availability

All supporting data to be made available upon request.

## Supplementary Materials

Supplementary 1 Supplementary Table 1: primer list.

Supplementary 2 Supplementary Table 2: differentially expressed genes between 4-week-old WT and KO CMs.

Supplementary 3 Supplementary Table 3: differentially expressed genes between KO saline-treated and WT saline-treated CMs.

Supplementary 4 Supplementary Table 4: gene ontology terms in upregulated genes in the KO saline compared to WT Saline.

Supplementary 5 Supplementary Table 5: gene ontology terms in downregulated genes in the KO saline compared to WT Saline.

Supplementary 6 Supplementary Table 6: differentially expressed genes between KO doxorubicin-treated and KO saline-treated CMs.

Supplementary 7 Supplementary Table 7: differentially expressed genes between WT doxorubicin-treated and WT saline-treated CMs.

Supplementary 8 Supplementary Table 8: commonly dysregulated genes and gene ontology terms in KO DXB- and WT DXB-treated cells.

Supplementary 9 Supplementary Table 9: gene ontology terms in the genes only dysregulated in the KO DXB-treated CMs.

Supplementary 10 Supplementary Table 10: differentially expressed genes between KO doxorubicin-treated and WT doxorubicin-treated CMs.

Supplementary 11 Supplementary Table 11: gene ontology terms in upregulated genes in the KO doxorubicin-treated CMs compared to WT doxorubicin-treated CMs.
